# Bullous Skin Rash: A Rare Case of Palbociclib-Induced Dermatological Toxicity

**DOI:** 10.7759/cureus.10229

**Published:** 2020-09-03

**Authors:** Noman Ahmed Jang Khan, Mohamed Alsharedi

**Affiliations:** 1 Hematology and Medical Oncology, Joan C. Edwards School of Medicine at Marshall University, Huntington, USA

**Keywords:** palbociclib, blistering skin disease

## Abstract

Palbociclib is an FDA-approved cyclin-dependent kinase inhibitor to treat hormone-positive, HER2-negative metastatic breast cancer. Severe skin toxicities are rare but important adverse events associated with these agents. Early detection of severe forms of skin lesions is crucial to permit the immediate discontinuation of palbociclib in order to avoid unacceptable risk level in the form of severe cutaneous toxicities like Steven-Johnson Syndrome. In such cases, palbociclib should be abruptly discontinued and an early aggressive support should be initiated. We here present a case of 50-year-old Caucasian female, who developed acute onset blistering skin lesions one to two weeks after she was started on palbociclib. We sought to report this case given the unusual toxicity and to emphasize the importance of identifying the acute onset of blistering skin lesions, regardless of their extension, should prompt awareness of their potential severity.

## Introduction

Palbociclib is a well-known cyclin kinase 4/6 inhibitor used in metastatic hormone receptor-positive breast cancer. It is most commonly used as a first-line treatment in conjunction with aromatase inhibitors. Neutropenia, anemia, thrombocytopenia, nausea, diarrhea, and skin rash are some of the reported adverse effects (AEs). The most common skin toxicities are grade 1, ranging from mild urticaria to pityriasis. More severe (grade 3) skin rash was less than 1% so far in the clinical literature as we found only one case of severe cutaneous toxicity presented as Stevens-Johnson syndrome [1]. Acute bullous skin rash is very rare and warrants immediate discontinuation of palbociclib. We report this case of acute bullous skin rash in a patient who was recently started on palbociclib for metastatic hormone receptor-positive breast cancer.

## Case presentation

**Figure 1:**
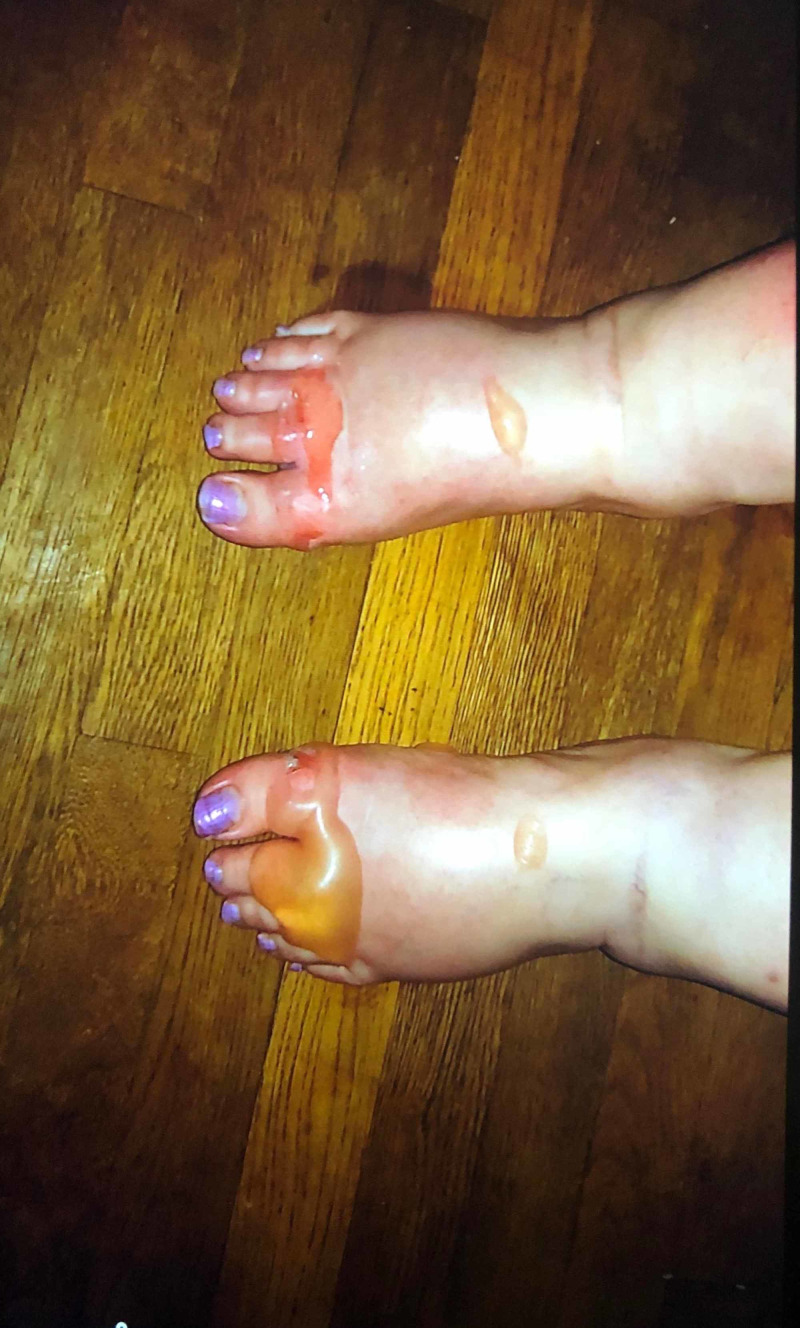
Blistering skin lesions on the dorsal surface of both feet.

**Figure 2:**
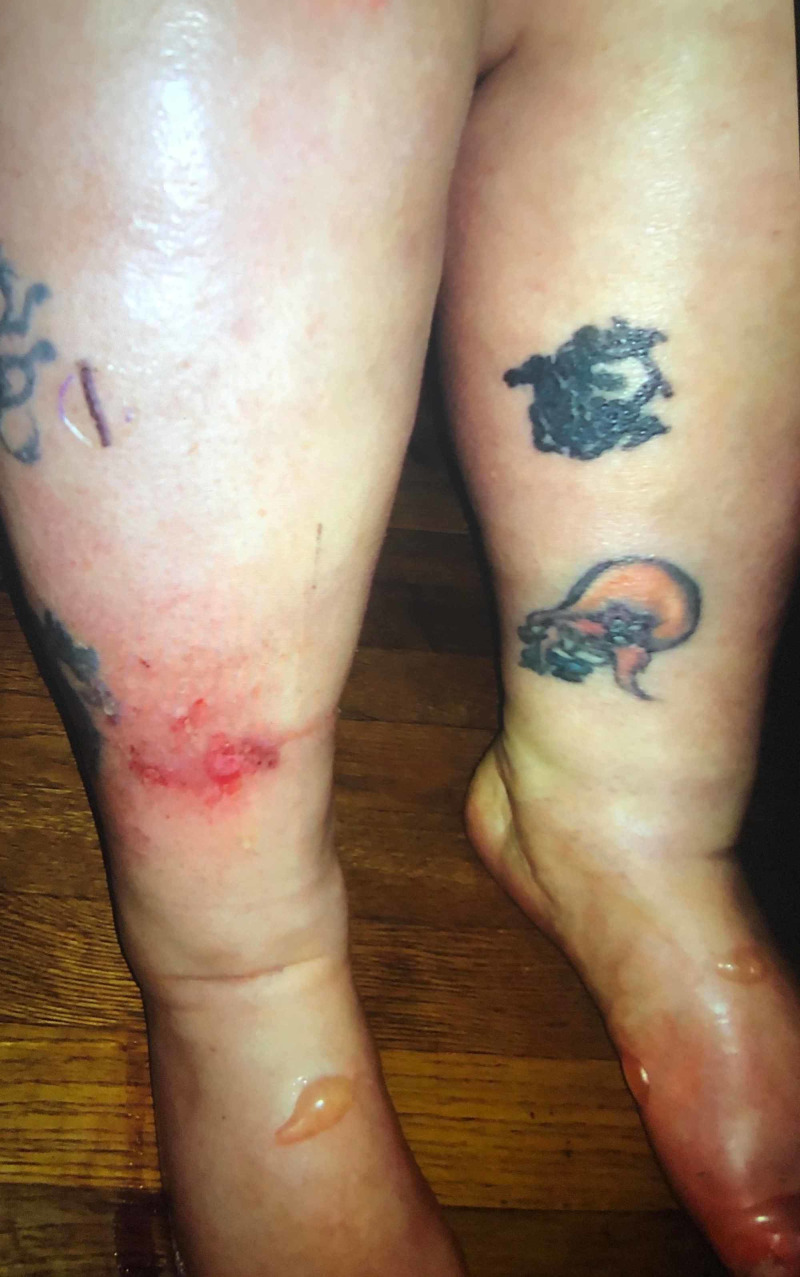
Blistering skin lesions on bilateral feet. One ruptured lesion also seen on the lower anterior right shin

A 50-year-old female was diagnosed with metastatic hormone receptor-positive breast cancer. She initially presented with hip pain on the right side. MRI of the hip revealed multiple foci of signal abnormalities in the pelvic bones and bilateral femurs. She underwent a right-sided hip replacement, and the bone biopsy revealed a metastatic invasive ductal carcinoma with estrogen receptor (ER) 80%, progesterone receptor (PR) 15.3%, and human epidermal growth factor 2 (HER2) receptor not overexpressed. Further workup included CT of the chest/abdomen/pelvis and an MRI of the cervical, thoracic, and lumbar spine, revealing extensive bony metastasis throughout the spine and pelvis but no evidence of visceral metastasis. Since the patient was still premenopausal at the time of diagnosis, she was started on tamoxifen along with monthly gosarelin injections for ovarian suppression. After her postmenopausal status was confirmed via hormone levels, she was switched to the aromatase inhibitor anastrazole. After a month, the cyclin-dependent kinase (CDK) 4/6 inhibitor palbociclib was also added to the anastrazole. Two weeks later, she presented to her local oncologist office with a skin rash on her lower extremities. Upon physical examination, fluid-filled bullous lesions were noticed bilaterally on the dorsal surface of her feet with very few ruptured lesions present on the right foot and shin (Figures 1, 2).

**Figure 3:**
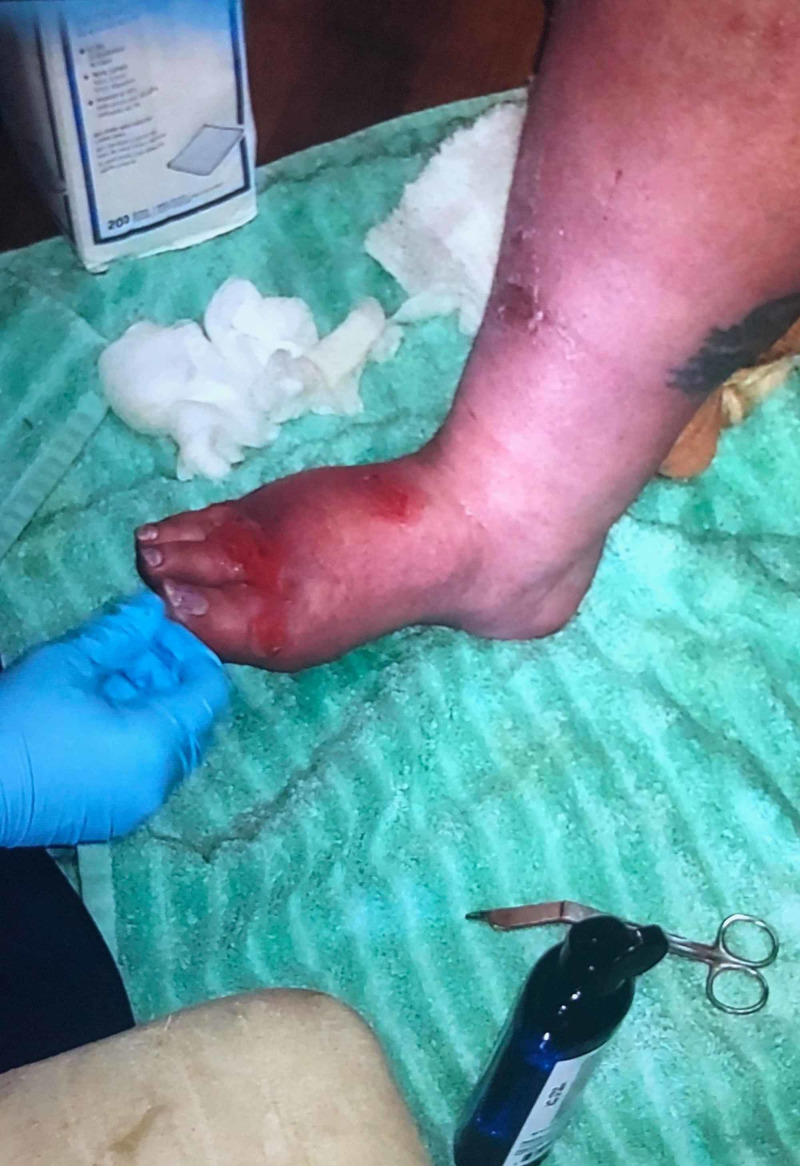
Improving blistering skin lesions seen on the right foot two weeks after discontinuation of palbociclib.

According to the patient, the lesions erupted around one week after she started palbociclib. The Nikolsky sign was negative. No evidence of oral mucosal involvement was seen. Palbociclib was immediately discontinued, and she was admitted to the hospital for close monitoring. No immunosuppressive agents, including topical corticosteroids, were administered. The bullous lesions started to erupt in the following days, and no new lesions were seen (Figure 3). She was eventually discharged from the hospital. Since palbociclib was the only medication started around the time of bullous eruptions and improvement occurred after abrupt discontinuation, we strongly believe these skin lesions were associated with it. Biopsy of the lesions could not be obtained due to personal reasons of the patient. 

## Discussion

Activation of CDK promotes cell cycle progression leading to abnormal cell growth. First-generation CDK inhibitors were initially studied for metastatic hormone receptor (HR+) breast cancer, but due to their pan-CDK inhibitions, numerous unacceptable toxicities were reported and their use was abandoned [2]. Therefore, selective CDK 4/6 inhibitors, such as palbociclib, abemaciclib, and ribociclib were introduced [3]. All CDK 4/6 inhibitors in combination with aromatase inhibitors are standard-of-care first-line agents in metastatic HR+ breast cancer [4-6].

CDK 4/6 inhibitors are associated with numerous AEs. Cytopenias are one of the most commonly reported AEs with these agents [7]. Palbociclib and ribociclib cause neutropenia in almost 70% of cases. Abemaciclib is very frequently associated with significant diarrhea [3], whereas ribociclib is known to cause QTC prolongation [8]. Skin reactions have been reported with CDK 4/6 inhibitors but are usually milder and do not warrant discontinuation of the treatment. However, bullous pemphigoid (BP) or bullous skin lesions have never been reported with these agents in literature. BP is an immune-mediated skin reaction that has been linked to several drugs, including mefenamic acid [9], etanarcept [10], terbinafine [11], and ampicillin [12]. More recently, dipeptidyl peptidase IV inhibitors (gliptins) have been found to cause BP [13]. Full-blown BP is usually characterized by the presence of tense blistering lesions with desquamative gingivitis involving the mucosal surfaces, but localized lesions are also seen in 10% to 20% of cases [14]. Suspicious lesions should be biopsied and evaluated via direct immunofluorescence (DIF), which is the most sensitive test for bullous autoimmune diseases [15].

As mentioned in the case history, a perilesional biopsy could not be obtained in our patient, but classically appearing tense blisters without any other obvious predisposing risk factors except for recent initiation of palbociclib were highly suggestive of drug-induced localized autoimmune bullous disease. Identification of the offending medication is crucial and warrants immediate discontinuation of the agent. Studies have shown that localized bullous disease could be an early manifestation of an advanced and life-threatening BP; therefore, early identification of these lesions is essential [14]. Palbociclib was immediately discontinued in our patient.

High-potency topical corticosteroids are the mainstay of treatment. In one study, high-intensity topical steroid therapy was found to be superior to systemic steroids [16]. In some cases, glucocorticoid-sparing agents, such as azathioprine, methotrexate, or mycophenolate mofetil, could also be used [17]. Considering the subacute nature of disease in our patient, she was closely monitored without requiring any immunosuppressive agents. She showed good clinical improvement.

## Conclusions

Palbociclib is a well-known CDK 4/6 inhibitor used widely in metastatic HR+ breast cancer. It is generally well tolerated, with cytopenias as the most commonly reported AEs. Grade 3 skin toxicities are very rare, but early identification of blistering skin lesions and their prompt discontinuation can prevent life-threatening disease and significant patient comorbidity.
